# Personal Hygiene and Physical Training for Women

**Published:** 1911-05

**Authors:** 


**Personal Hygiene and Physical Training for Women.** By **Anna M. Galbraith,** M.D., Fellow of the New York Academy of Medicine. 12 mo of 371 pages, with orignal illustrations. Philadelphia and London: W. B. Saunders Company. 1911. (Cloth, $2.00, net.)

The purpose of the author is "to present in a clear and concise manner the fundamental physiological laws of personal hygiene, with directions for the development of the body, so that the tissues will be placed in the best possible condition to resist disease." What could be more elemental, practical and modest than this, and how widely this differs from the first and second duties of woman as set forth in the catechism?

The book consists of nine chapters and its contents will be indicated by glancing at their subjects: "Hydrotherapy ; The care of the skin and its appendages; The digestive system and the maintenance of good digestion; The respiratory and circulatory systems, the kidneys; The nervous system as the balance of power in the body; The hygiene of the mind and its relation to the physical health; dress the fundamental cause of woman's deterioration; Physical training the key to health and beauty; Symmetric development, good carriage and grace of motion through gymnastics and athletics."

Those of our readers (and we admit they are in the majority) who hold that the personal hygiene of woman is exclusively a question of physics will be pleased with and instructed by this work. The illustrations of the book are quite unusual and delightful. H. R. H.

